# Recruitment of young adults for weight gain prevention: randomized comparison of direct mail strategies

**DOI:** 10.1186/s13063-016-1411-4

**Published:** 2016-06-08

**Authors:** Melissa M. Crane, Jessica Gokee LaRose, Mark A. Espeland, Rena R. Wing, Deborah F. Tate

**Affiliations:** Gillings School of Global Public Health, University of North Carolina Chapel Hill, Rosenau Hall, Box 7440, Chapel Hill, NC 27599-7440 USA; Division of Epidemiology & Community Health, School of Public Health, University of Minnesota, 1300 S. 2nd Street, Suite 300, Minneapolis, MN 55454 USA; Virginia Commonwealth University, School of Medicine, One Capitol Square, 9th floor, Richmond, VA 23298 USA; Wake Forest University School of Medicine, Medical Center Boulevard, Winston-Salem, NC 27157 USA; The Miriam Hospital/Weight Control and Diabetes Research Center, 196 Richmond Street, Providence, RI 02903 USA; Department of Psychiatry and Human Behavior, Warren Alpert Medical School of Brown University, Providence, RI USA

**Keywords:** Recruitment, Young adults, Weight gain prevention, Men

## Abstract

**Background:**

Recruiting young adults (ages 18–35 years) into weight gain prevention intervention studies is challenging and men are particularly difficult to reach. This paper describes two studies designed to improve recruitment for a randomized trial of weight gain prevention interventions. Study 1 used a quasi-experimental design to test the effect of two types of direct mailings on their overall reach. Study 2 used a randomized design to test the effect of using targeted messages to increase recruitment of men into the trial.

**Methods:**

For Study 1, 60,000 male and female young-adult households were randomly assigned to receive either a recruitment brochure or postcard. Visits to recruitment websites during each mailing period were used to assess response to each mailing. Study 2 focused on postcard recruitment only. These households received either a targeted or generic recruitment postcard, where targeted postcards included the word “Men” in the headline text. Response rates to each type of card were categorized based on participant report of mailing received.

**Results:**

The reach of the postcards and brochures were similar (421 and 386 website visits, respectively; *P* = 0.22). Individuals who received the brochure were more likely to initiate the online screener than those who received a postcard (*P* = 0.01). In Study 2, of those who completed the telephone screening, 60.9 % of men (n = 23) had received the targeted postcard as compared to the generic postcard (39.1 %, *P* = 0.30). The reverse was true for women (n = 62, 38.7 vs. 61.3 %, *P* = 0.08).

**Conclusions:**

These studies suggest there was little difference in the reach of postcards versus brochures. However, recipients of brochures were more likely to continue to the next stage of study participation. As expected, men’s response to the weight gain prevention messages was lower than women’s response; but using targeted messages appears to have modestly increased the proportion of male respondents. These studies add to the limited experimental literature on recruitment messaging and provide further indication for using targeted messages to reach underrepresented populations while providing initial evidence on the effect of mailing type on message reach.

**Trial registration:**

The Study of Novel Approaches to Weight Gain Prevention was registered with ClinicalTrials.gov (identifier: NCT01183689) on 13 August 2010.

**Electronic supplementary material:**

The online version of this article (doi:10.1186/s13063-016-1411-4) contains supplementary material, which is available to authorized users.

## Background

Young adulthood has been identified as a high-risk developmental period for weight gain and a potential time for weight management intervention [1, 2]. Weight gained during this period averages approximately 13 kg [3] and is associated with a doubling in the prevalence of obesity between the early 20s and the late 20s or early 30s [4]. Across racial and sex subgroups, weight gained during this period is also associated with developing poorer cardiovascular health markers, including increased blood glucose and systolic and diastolic blood pressure [5]. Despite this, young adults report minimal concern about gaining weight. In a recent survey, college freshmen reported that they would need to gain an average of 5 to 8 % of their body weight (3.1 to 6.2 kg) before they took action to reverse the weight gain [6]. Proven approaches to prevent weight gain in young adults are not readily available and are the subject of clinical trials seeking to reverse those trends, including those funded through the Early Adult Reduction of weight through LifestYle Intervention (EARLY) cooperative agreement sponsored by the United States National Heart, Lung, and Blood Institute (NIH 5U01HL096720) [7]. The task of recruiting this age group into these trials has proven somewhat challenging [8].

Direct mailing is a commonly used mode of recruitment into randomized trials due to its broad reach and relatively low cost [9]. Additionally, this approach can be particularly effective when trying to reach underrepresented populations via purchasing targeted lists from sources such as magazine subscription lists or lists of registered drivers in the target area [10]. Direct mail has been used successfully to recruit both adults and young adults into weight loss programs [11, 12]. Although direct mailings are often used, there is little evidence to guide researchers when developing the messages to use when recruiting for randomized trials. Using health communication theory, there are aspects of direct mailings that can be experimentally tested in order to create more effective direct mail recruitment materials for trials.

One aspect of recruitment messages that needs to be examined is how to maximize the persuasiveness of the messages. The Elaboration Likelihood Model postulates that persuasive messages are evaluated via central or peripheral routes. In central processing, the recipient takes an active role considering message features such as the message’s source (expert versus non-expert), level of trustworthiness, and the number and quality of the arguments included in the message, described together as the “quality” of the argument or message. On the other hand, in peripheral processing, the recipient is less engaged and focuses on less meaningful aspects of the message [13]. The primary determinations for whether a message is processed via peripheral versus central processing are the motivation of the recipient to engage with the messages and their ability to do so. If a message is more personally relevant, the recipient’s motivation to process the message is increased and the recipient is more likely to use central processing. It stands to reason that considering participation in a weight management program (either weight loss or weight gain prevention) would be a topic that could have a great impact on a person’s life, involving daily changes to eating and exercise behaviors. Therefore, developing and testing higher quality messages that will be positively evaluated during central processing may be important in recruiting difficult-to-reach populations into research trials.

To date, no studies have specifically manipulated the quality of the message used for recruitment into weight management studies. Gerace et al. [14] conducted the closest comparison found in the published literature where they experimentally compared the amount of information, or number of arguments, included in the recruitment mailing. The number of arguments included in a message is one aspect of message quality, but if the quality of the arguments is low, number becomes less important [13]. In the Gerace et al. [14] study, the additional length of the message did not improve recipient response. Given the lack of research on message quality on recruitment yield, it is important to test whether the quality of the message can influence its persuasive qualities and result in recipients seeking more information about or enrolling in the study. This question was assessed in Study 1, where we tested whether varying the “quality” of the messages by manipulating the amount and type of information provided would improve the yield of young adults seeking information about a weight gain prevention trial.

### Recruiting men into weight gain prevention

While all young adults are challenging to reach with messages of weight gain prevention, men appear to be particularly difficult. Men report needing to gain 6.2 kg before they would change their behavior as compared to women’s reported 3.1 kg. Further, significantly fewer men (17 %) than women (61 %) report interest in participating in a weight gain prevention program [6]. Qualitative evidence suggests that young men perceive that there is societal acceptability for men to gain weight with age, but that the same does not hold true for women. These perceptions among young men have been apparent in studies that have focused on weight control using diet and physical activity among 18- to 35-year-olds. In a pilot study for the current research, only 2 % of participants were men [15]. Similar results were found when looking at studies of weight loss among adults [16] as well as for weight loss targeted toward young adults [17, 18]. In addition to men’s low interest in weight control, the low percentage of men in the programs may be due to the perception that these programs are designed specifically for women [19]. To overcome this perception, it may be beneficial to use message targeting to increase the likelihood that men will identify with recruitment messages, pay attention to the recruitment message and, in turn, express interest in the program.

Using targeted language and images is one well-researched approach to improve the reach of health communications within specific subpopulations [20]. Targeting uses group identification, often race or ethnic group, to increase the personal relevance of the message to the recipient. Increased personal relevance is hypothesized to increase the attention given to the message and increase the cognitive processing of the message [13]. There is experimental evidence from two studies that targeting recruitment messages increases interest in participation in weight loss programs. The first study by Kiernan et al. [21] found that including targeted health risk information in direct mailings for recruitment for a weight loss program for Hispanic employees increased response rates from 6.5 to 9.1 %. Although this increase did not reach statistical significance, the authors suggest that a meaningful trend was evident but the study was not adequately powered to detect the sizeable increase due to their relatively small sample (*n* = 561). In a more recent study, Brown et al. [22] compared using direct mailing of recruitment information sent to Hispanic women that contained generic health information, targeted health information, personalization of the letter, or both targeting and personalization. In this study, women who received the targeted mailing were more likely to respond than women who received the generic information. There was no effect found for personalizing the letter nor was there a targeting by personalization effect. These studies suggest that targeting may be a useful tool for recruitment for weight loss studies; however, no studies have experimentally tested targeting recruitment messages for other subgroups beyond Hispanic populations.

The purpose of this is paper is to report the results of two studies designed to evaluate direct mail recruitment efforts from one site of the multicenter trial of Study of Novel Approaches to Weight Gain Prevention (SNAP). While many modes of recruitment were used during recruitment for this study [8], direct mailing provides a more accurate estimate of the number of recipients of a message as compared to estimates associated with other modes of study advertising (e.g., television, newspapers, flyers, email, etc.). Direct mail provides the benefit of a clear “denominator” for testing the reach of the messages and was therefore chosen for use in these studies. In Study 1, we compared the relative reach of a shorter, potentially lower quality message delivered via a postcard to a longer and higher quality message provided via a tri-fold brochure. We sought to test whether the quality of the message would influence participant response using a quasi-experimental design. We hypothesized that the message delivered via the brochure, which included a longer and more detailed description of the study staff expertise and benefits of participating in the study, would generate a greater response as compared to the brief message delivered via the postcard. In Study 2, we compared generic messages focused on weight gain prevention to messages that targeted men using a randomized experimental design. We hypothesized that a greater proportion of male respondents would report receiving a postcard that included targeted communication compared to one with generic communication.

## Methods

### Main study

The studies presented here use data collected during the direct mail recruitment at University of North Carolina for the SNAP. Full details of the study are available in Wing et al. [23]. Briefly, SNAP is a multicenter, NIH-funded randomized trial comparing the effect of two approaches to weight gain prevention among normal and overweight (body mass index (BMI), 21.0–30.0 kg/m^2^) young adults (18–35 years). The approaches being evaluated include self-regulation with Small Changes or self-regulation with Large Changes as compared to a minimal intervention control [23].

Overall, 599 young adults (*n* = 307, North Carolina and *n* = 292, Rhode Island) were randomized into the SNAP study across the two clinical research sites. Recruitment efforts varied by research site and have been described in detail elsewhere [8]. In addition to direct mailings, recruitment efforts included community events; internet advertisements or website postings, mass emails to listservs or purchased email lists, and newspaper, television, and radio advertisements. Messages for recruitment were developed based on results from focus groups conducted with young adults about their views on weight and the potential for weight gain [8]. Across both clinical centers, direct mail was the method through which the majority of participants were recruited.

All modes of recruitment directed potential participants to a study website to begin participation. Three identical websites were created, where only the web addresses were different: a website to use for general recruitment efforts (www.snapstudy.org) and two websites that were developed specifically for these direct mail recruitment studies described here (snap4men and snapaverage, described below). All three websites provided the same description of the SNAP study, including eligibility criteria, the purpose of the study, a BMI calculator, and a link to an online pre-eligibility screening form. Individuals interested in participating were instructed to access the online screening form using a link on the website. This link took visitors to a secure website to complete the pre-screening form. The online screening forms were assessed for initial eligibility and participants who met the age and BMI criteria were then contacted via telephone to further determine eligibility. As described by Tate et al. [8], 33.9 % of participants who completed the online screener were pre-eligible and completed the telephone screen. The final recruitment step included attending an in-person study orientation session where study procedures were described. Participants who chose to take part in the study then provided informed consent to take part in the trial. All study procedures were approved by the University of North Carolina non-biomedical institutional review board (IRB Study number: 07-1783).

### Study 1

Data for this analysis come from recruitment from the North Carolina clinical research site only. A targeted mailing list of 60,000 names and addresses of male and female head-of-households between the ages of 18 and 35 within 30 miles of the North Carolina clinical site was purchased from USA Data, Inc. The addresses were randomly assigned to receive a generic postcard (*n* = 15,000), a targeted postcard (n = 15,000), a generic brochure (n = 15,000), or a targeted brochure (n = 15,000; targeted and generic materials are described below). Randomization was completed using a random number generator and did not stratify on sex of the recipient. Although addresses were randomized to receive one of the four mailings during a single randomization, the brochures and postcards were mailed separately. The quasi-experimental analysis for Study 1 compares the 30,000 postcards sent in May 2011 and the 30,000 brochures mailed in December 2011. The mailings were sent separately to aid in recruiting cohorts to begin the study.

To analyze the reach of the mailings, website visits to the two websites associated with the mailings were recorded. IP addresses of all visitors to the websites were recorded and time stamped. Each visit was classified as including a click on the link to the pre-screening form or not. To assess independent visits to the websites, duplicate addresses were removed such that the earliest visit or the visit that contained a click on the screening form link were retained.

**Fig. 1:**
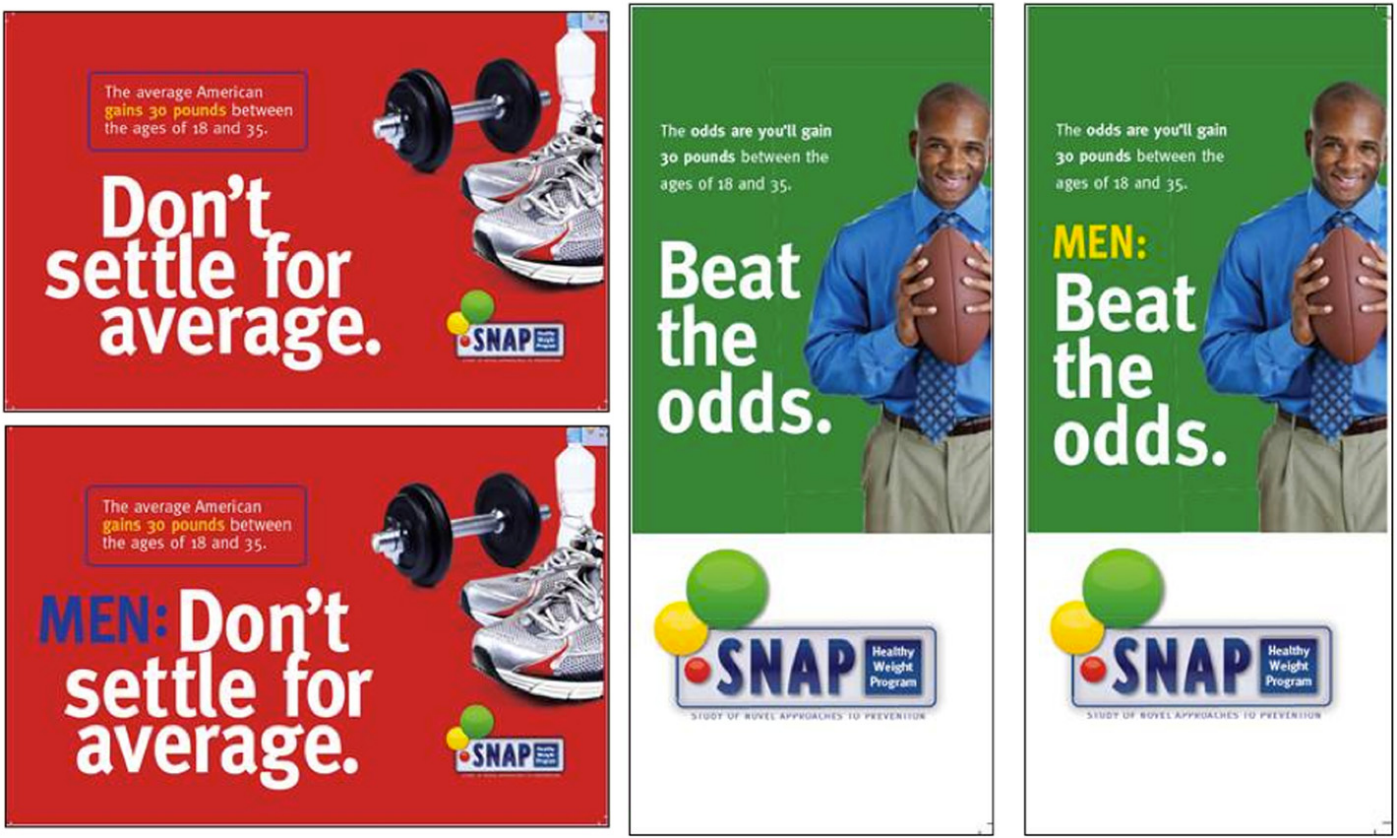
Front of recruitment materials. *Top left* Generic Postcard, *bottom left* Targeted Postcard, middle Generic Brochure, *right* Targeted Brochure

The postcards (216 mm × 139.5 mm; see Fig. 1 and Additional file 1) included a brief description of the SNAP study including the general purpose of the study, eligibility criteria, and study sponsors. The postcards were full-color, two-sided, and contained 156 words in the study description. The brochures (tri-fold, two-sided, full-color, 216 mm × 279 mm unfolded) contained the same information as the postcards but also included additional information hypothesized to make the message more persuasive. During formative work for this study, young adults in focus groups stated that the immediate benefits of participating in the study would be more persuasive than focusing on the longer-term benefits [8]. To address this, the brochures included a list of immediate benefits of participating in the study (including free personalized analysis of nutrition and physical activity). The brochures also included a description of the expertise of the study staff (i.e., expertise of weight control professionals (nutritionists, exercise physiologists, physicians, health educators, psychologists, nurses)) while the postcard used a more general description (i.e., team of professionals). The brochure also included a participant testimonial and a lengthier description of why weight-gain prevention is important. The brochure contained 440 words in the study description.

To analyze the reach of these two types of direct mailings, website visits associated with the mailings were recorded during the two 8-month recruitment periods: May 2011 to December 22, 2011, and December 23, 2011, to August 2012. Binomial proportion tests were used to compare the number of website visits by mailing type. The null hypothesis tested was that the proportion would be equal across both mailing types. To test whether mailing type influenced the rate at which participants continued to the online screener, χ^2^ analysis was used.

### Study 2

Study 2 compared the effect of male-targeted messages on increasing male response to recruitment messages. The headline text of the generic postcards featured a social norms-based message “Don’t Settle for Average. The average American gains 30 pounds between the ages of 18 and 35” (Fig. 1). For the targeted postcards, this phrase was changed to “Men: Don’t Settle for Average…” Targeted and generic postcards directed interested recipients to separate, but identical websites, with unique web addresses (generic postcards directed participants to snapaverage while targeted postcards directed participants to snap4men). Both websites were identical and provided links to initiate the screening process. Both the postcards and websites stated that the study was recruiting men and women for study participation^1^.

Participants who were initially eligible based on the online screening form were contacted via telephone to complete the study eligibility screening. During the telephone screening, participants were asked to indicate how they heard about the study. Those who indicated they heard about the study via a postcard were asked to indicate which website they visited (either snapaverage or snap4men). This served as the self-report of the type of message received. The names and addresses of participants who received a postcard were compared to the names and addresses to which the postcards were sent. This served as a confirmation for the classification of type of message sent (generic or targeted). Among participants for whom both self-reported and confirmed mailing information was available, 86 % correctly reported their direct mailing message. In the absence of confirmed mailing information (*n* = 12, 14 %), self-reported mailing type was used to classify respondents.

To test whether the targeted messages increased the proportion of male respondents, a χ^2^ analysis was used. For analyses that compared counts across two levels (i.e., compared number of website visits), binomial proportion tests were used. In each case, the null hypothesis tested was that the proportion would be equal across both groups. χ^2^ analysis was used to test whether final randomization rates varied by sex or postcard type received.

## Results

### Study 1

**Fig. 2:**
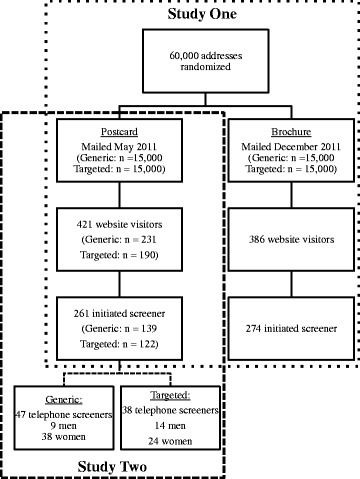
Website visits and telephone screenings by message and mailing type

As shown in Fig. 2, there were 807 independent visits to the two websites associated with the direct mailings, a response rate of 1.3 %. Website visits during the period associated with the mailing of the postcards made up 52.2 % of website visits (*n* = 421) while the period for the brochures represented 47.8 % of visits (*n* *=* 386, *P* = 0.22). Of the 807 visits to the websites, 535 (66.3 %) visitors initialized the online screening form. Those who were sent a brochure were significantly more likely to initialize the online screener process than those who received a postcard (71.0 vs. 62.0 %, OR = 1.50, 95 % confidence interval (CI), 1.12–2.01, *P* = 0.01).

### Study 2

In response to the postcards, there were 421 visits to the recruitment websites (Fig. 2). The website associated with the targeted postcards (i.e., snap4men) received significantly fewer visits (n = 190, 45.1 %) than the generic website (i.e., snapaverage; *n* = 231, 54.9 %, *P* = 0.05). There was no difference in initialization rates of the online screener by website: 64.2 % of those receiving the targeted vs. 60.2 % of generic initiated the study enrollment screener (OR = 1.11, 95 % CI, 0.87–1.43, *P* = 0.40).

After initial online screening, a telephone screening was conducted. A total of 85 respondents (23 male, 62 female) indicated that they received a postcard as their mode of recruitment. There was no difference in the number of screenings completed by those receiving targeted (*n* = 38, 44.7 %) versus generic postcards (*n* = 47, 55.3 %, *P* = 0.33). Of the 23 men, 60.9 % were responding to the targeted mailing compared to 39.1 % for the generic mailing (*P* = 0.30). The reverse was true for women (Targeted, 38.7 %; Generic, 61.3 %; *P* = 0.08). Together, there was a trend for the sex of respondents to be associated with the type of mailing received (OR = 2.46, 95 % CI, 0.92–6.57, *P* = 0.07).

Among eligible participants recruited by postcards (n = 85), 30 were randomized into the SNAP study. There was no difference in the proportion of each sex randomized into the SNAP study (Men, 39.1 %, *n* = 9; Women, 33.9 %, n = 21; OR = 1.26, 95 % CI, 0.47–3.37, *P* = 0.65). Similarly, there was no difference in percent of participants recruited by targeted (39.5 %, n = 15) or generic postcards (39.1 %, n = 15) who were randomized into the study (OR = 0.72, 95 % CI, 0.29–1.76, *P* = 0.47).

## Discussion

This study used quasi-experimental (Study 1) and experimental (Study 2) designs to compare the effect of varying the quality of the message (postcard versus brochure) and the type of message (targeted versus generic) to recruit normal and overweight young adults into a randomized controlled trial of methods for weight gain prevention. The results indicate that the brochures yielded a comparable response rate to the postcards and appear to have led to a higher rate of initializing enrollment via the initial online screening form. Further, using targeted postcard communication may have increased the proportion of male respondents relative to generic postcard communication (60.9 vs. 39.1 %), although this did not reach statistical significance (*P* = 0.30).

Our hypothesis that a more persuasive message delivered by the brochures would lead to greater response than the shorter message delivered via postcards was partially supported. While the brochures contained all of the information included in the postcards, they included additional information such as the benefits of participating in the SNAP study. Formative work with young adults suggested that focusing on immediate benefits would make the program more appealing than focusing on long-term benefits alone and these data appear to support this assertion. Many young adults do not see themselves at risk for gaining weight [5]; therefore, explicitly listing the immediate benefits of participating in the program, such as personalized nutrition analysis, may make participating in the study more appealing beyond the potential distal benefits for their weight and health. By presenting these benefits in the mailing, rather than relying on participants to identify them while visiting the website, the messages may have been more persuasive and the positive evaluation of the program may have been enhanced.

Additionally, the brochures may have been viewed as more persuasive due to the inclusion of more information about the expertise of those conducting the study and delivering the intervention. As put forth in the Elaboration Likelihood Model, messages from a trusted and expert source are more persuasive than messages that are perceived as less trustworthy [13]. The brochures included a statement that focused on describing the expertise of the university-employed interventionists and study investigators while postcard included less information on the staff’s expertise. This description of study staff may have served to increase the perceived trustworthiness of the source and increased the interest in the study. This effect, though not able to be tested in this study, may have been particularly relevant to men who have reported seeking information regarding weight loss only from what they perceive as trustworthy sources [24]. Similarly, including participant testimonials may have increased participants’ interest by increasing social norms for participation in the study.

In addition to these explanations, it is possible that the format of the brochure, which included more visuals and more textual information overall, may have been more persuasive than the postcards, regardless of the type of message included. Despite these limitations, the comparison between the postcards and the brochures represents a realistic question that researchers recruiting for studies may face: which type of direct mailing will be more effective? Using communication theory, we developed the brochures to include a higher quality message than the postcards. Future studies will need to test whether matched types of direct mailings with differing messages would yield similar results to those reported here.

Our hypothesis in Study 2, that men would be more likely to respond to a message targeting men, was also somewhat supported. This result is consistent with the findings reported by Kiernan et al. [21] and Brown et al. [22], both of which found that minority recipients who received a targeted recruitment letter were more likely to respond than those who received a generic letter. However, in both the current study and the previous studies, response remained low among the targeted groups. If it is assumed that approximately half of the addresses randomized to receive a postcard in the current study belonged to men, there were 15,000 potential male recipients of the mailings. Only 23 men completed the telephone screening process: a 0.15 % response rate. While this gives a sense of the response rate, it is important to note that a significant portion of potential participants were deemed ineligible prior to the phone screen and could not be included in the response rate reported here [8]. However, even with this limitation, this response rate is lower than that seen in Brown’s study (0.8 %), which also utilized community-based recruitment [22]. In response to all mailings, the response rate as measured as the number of online screeners initiated was 0.9 %. The lower response rate among men, along with the overall low response rate in this study, further emphasizes the challenge of promoting weight gain prevention among young adults for whom concern about weight gain may not be a priority.

One argument that may arise against using targeted recruitment messages is a reduced overall response rate. While the results of this study support this concern, it also demonstrated its minimal actual impact. Although the targeted messages did yield a lower response rate to the study website (45.1 % of website visits) compared to the generic message (54.9 % of visits), there were no differences in the proportion that started the online screener by type of recruitment message. However, as is common in programs focused on weight management [25], women were still overrepresented as compared to men. Further, although the targeted mailing explicitly mentioned men in the headline and the study web address, the study description stated that the study was for men and women, and many women responded. In fact, 38.7 % of female respondents recruited via a postcard were responding to the targeted message. Therefore, although the targeted mailing likely deterred some women, others overlooked this targeting and initiated the screening process.

This paper is unique in its use of a randomized comparison of recruitment messages and quasi-experimental comparison of mailing types. This contributes to the experimental literature on recruitment techniques for clinical trials, which currently contains mostly descriptive studies rather than experimental evidence. By directing interested recipients to separate but identical websites, we were able to assess the reach of each message objectively. Participants were also able to recall which website they visited with a high level of accuracy. This method of tracking message reach could be a useful technique for monitoring recruitment techniques and messages in other studies, provided that website addresses are designed to be easily remembered. Finally, this study tested the effects using direct mail, a commonly used and cost-effective recruitment strategy. This approach to reaching young adults was the most effective technique for recruitment in both the SNAP study as well as another weight loss program for young adults [12]. In SNAP, other techniques that were used included emailed, television, and radio advertisements. Direct mail was also one of the most cost-effective approaches for recruitment for SNAP [8], suggesting continued investigation of how to best develop direct mailing recruitment messages is a realistic and needed field of research.

The limitations of this study are related to the design as well as the response from recipients. The comparison between postcards and brochures is limited due to its quasi-experimental design and this study is unable to separate any effects that may be due to time of year from the effects of the mailing type itself. Specifically, there may be differences in responses due to month during which the mailing was received (May vs. late-December). Additionally, participants in the brochure condition had a longer time where they could have been exposed to the additional recruitment efforts that were ongoing. Repeated exposure to weight gain prevention messaging may have contributed to the sustained interest. Secondly, we were unable to test the effect of the type of mailing on increasing the proportion of male respondents due to prioritization of recruitment of men and minorities during brochure recruitment. This decision was made in order to recruit a more representative sample of participants for the main trial. This represents a challenge faced by any researchers embedding a recruitment study into ongoing recruitment for a clinical trial. Further, as described above, the response rates to this study were low: only 1.3 % of recipients visited one of the study websites. While this rate is lower than the 9.6–22.4 % response rate reported by Gerace et al. [14] when recruiting for weight loss among women 50–79 years old, it was greater than the 0.7 % response rate among Hispanic women reported by Brown et al. [22]. The low response in this study limited the power available to test for differences in our comparisons. However, this limitation further demonstrates the need to better understand how to reach potential research participants with health promotion programs.

## Conclusions

Recruiting adequate samples is necessary for the success of clinical trials. Despite this, there is little information in the published literature about how to successfully recruit for trials using data from randomized comparisons, especially for studies focused on health promotion behaviors such as weight gain prevention. This paper compared two aspects of direct mail recruitment that future program planners can use to expand their own recruitment efforts. The slightly higher cost of the brochures compared to the postcards (i.e., US$ 7914 vs. US$ 7422) may be offset by greater sustained response. The use of targeted messages did not increase the cost of the recruitment and is therefore an avenue for consideration even for studies with limited budgets.

There is ongoing and growing interest within the public health community in preventing negative health outcomes by preventing weight gain and building health-promoting habits before habits are well established in middle adulthood. Despite this interest within the research community, potential participants often remain disinterested in participating in trials focusing on this type of health promotion. This study provides initial work on how to better reach potential participants with these types of programs but it is clear that further research is needed to increase response rates within the target population. Additionally, there is a need for more research focused on the effect of recruitment messages on recruitment outcomes conducted in a more rigorous manner. Although reporting recruitment yields anecdotally can provide guidance into how to recruit research participants, there is a need for more studies that experimentally test recruitment methods and messages.

## Additional file

Additional file 1: Supplemental Content: Direct mailing materials. (PDF 2531 kb)

## Footnotes

1 Targeted and generic versions of the brochures were also developed; however, we were also unable to test the effect of the type of mailing on increasing the proportion of male respondents due to prioritization of recruiting men and minorities during brochure recruitment. During this focused recruitment, telephone screenings were conducted only for participants who identified as male or minority in the initial online screening form. Because recruitment source questions were included in the telephone screening, Study 2 used data only from the postcard recruitment period.
