# Supplementary material for: Recruitment of young adults for weight gain prevention: randomized comparison of direct mail strategies
Source: Trials. 2016 Jun 8;17:282. doi: 10.1186/s13063-016-1411-4 (PMC4897900; doi:10.1186/s13063-016-1411-4)
Supplement: Additional file 1: — Supplemental Content: Direct mailing materials. (PDF 2531 kb) [file 13063_2016_1411_MOESM1_ESM.pdf]

Generic Postcard Front

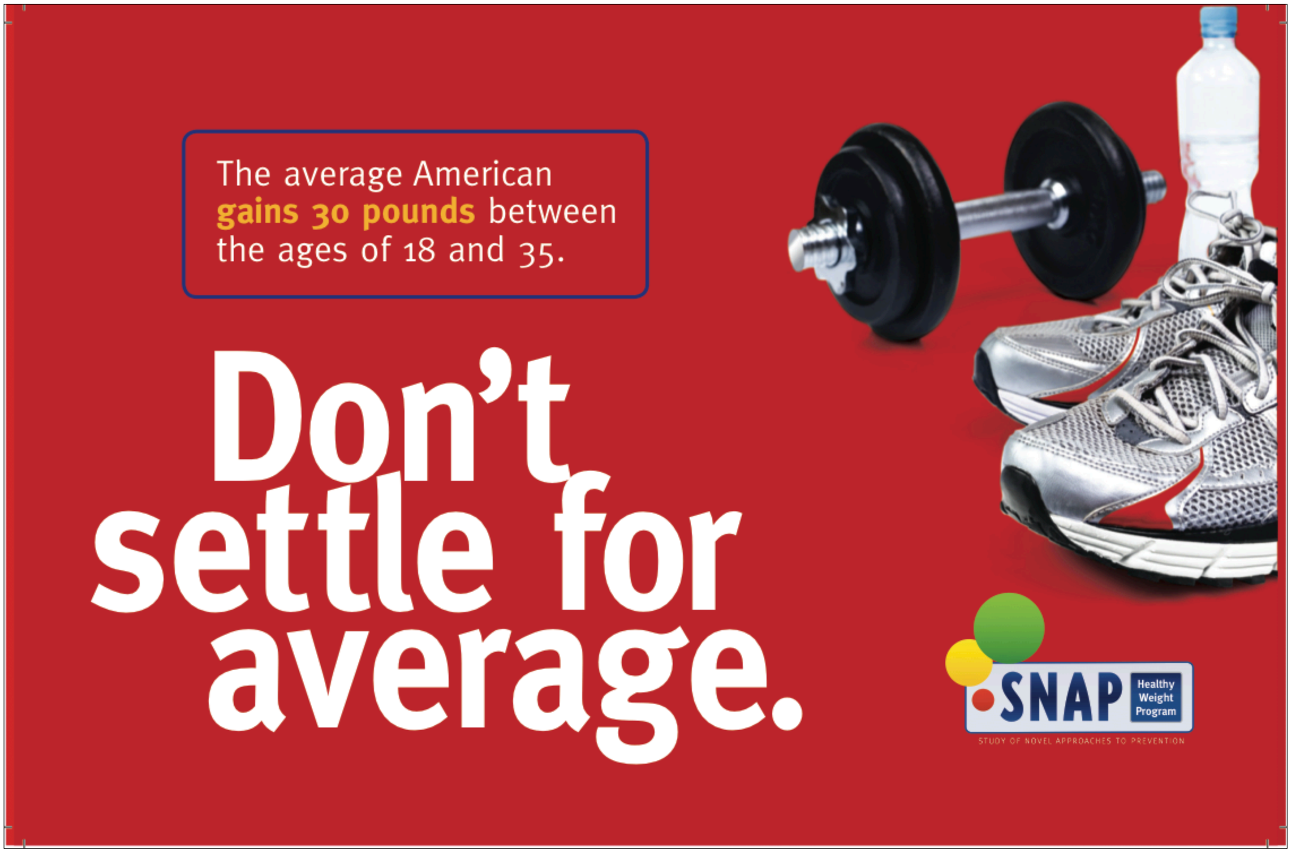

Generic Postcard Back

Want to fit in your current pant size in 15 years?

JOIN SNAP TODAY

The University of North Carolina Weight Research Program is recruiting for a no-fee research study in your area that provides 18-35 year old men and women with tools to prevent weight gain. You'll receive personalized feedback on your calorie expenditure, blood pressure, body fat percentage and cholesterol levels PLUS a detailed analysis of your diet and exercise patterns. Our team of professionals will help you create strategies for a healthier future. Modest weight loss can be expected.

If you are interested in participating, please go to www.snapaverage.org for more information and to see if you would be eligible to participate.

SNAP Healthy Weight Program
STUDY OF NOVEL APPROACHES TO PREVENTION

No fads, no gimmicks, no fees.
www.snapaverage.org
or call (919) 966-5852

THE UNIVERSITY of NORTH CAROLINA at CHAPEL HILL

National Institutes of Health

The SNAP Healthy Weight Program is Funded by the NIH and is Presented Locally by the The UNIVERSITY OF NORTH CAROLINA.

UNC Weight Research Program
www.uncweightresearch.org
1700 Martin Luther King Jr. Blvd., Suite 136
Chapel Hill, North Carolina 27599-7294

NONPROFIT ORG
US Postage
PAID

Targeted Postcard Front

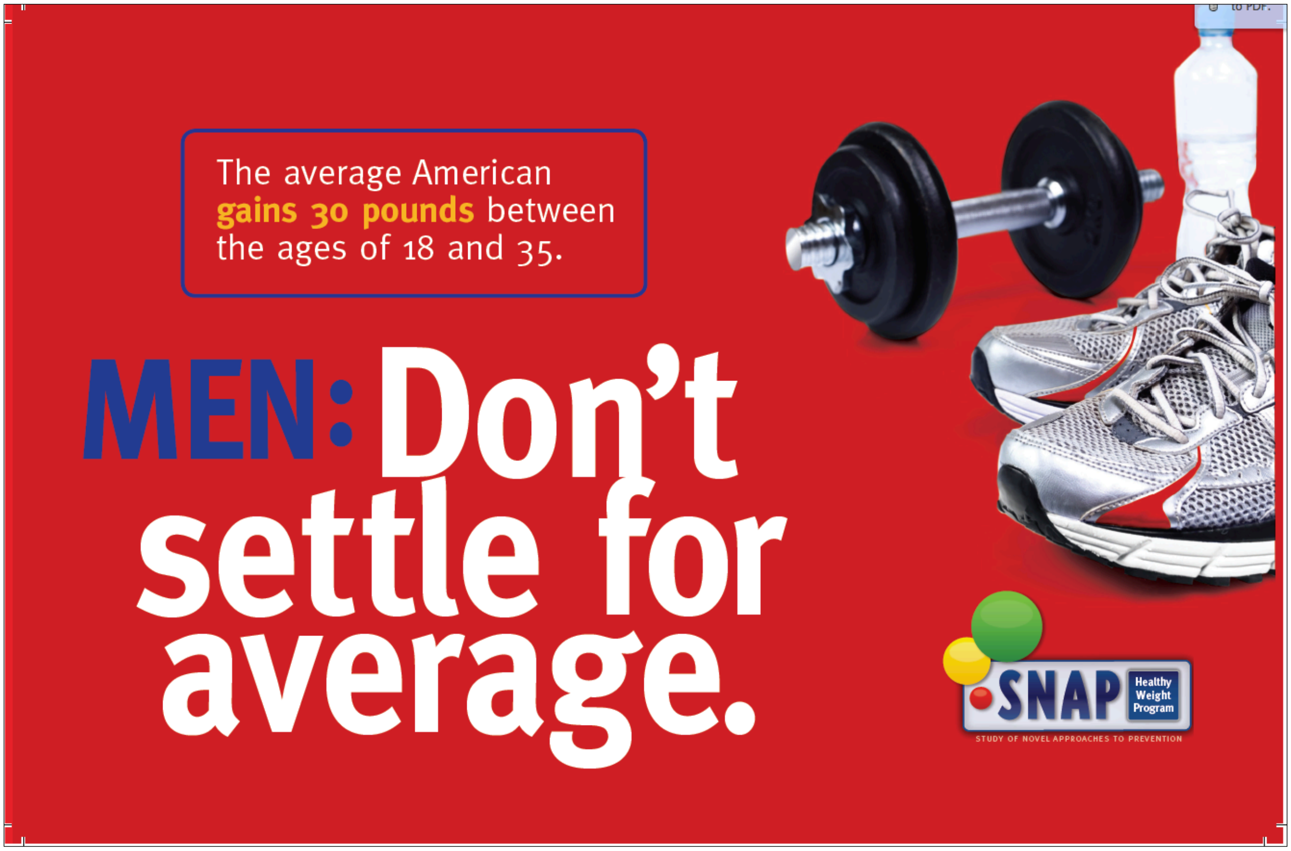

Targeted Postcard Back

Want to fit in your current pant size in 15 years?

JOIN SNAP TODAY

The University of North Carolina Weight Research Program is recruiting for a no-fee research study in your area that provides 18-35 year old men and women with tools to prevent weight gain. You'll receive personalized feedback on your calorie expenditure, blood pressure, body fat percentage and cholesterol levels PLUS a detailed analysis of your diet and exercise patterns. Our team of professionals will help you create strategies for a healthier future. Modest weight loss can be expected.

If you are interested in participating, please go to www.snapaverage.org for more information and to see if you would be eligible to participate.

SNAP Healthy Weight Program
STUDY OF NOVEL APPROACHES TO PREVENTION

No fads, no gimmicks, no fees.

www.snapaverage.org

or call (919) 966-5852

THE UNIVERSITY of NORTH CAROLINA at CHAPEL HILL

National Institutes of Health

The SNAP Healthy Weight Program is Funded by the NIH and is Presented Locally by the The UNIVERSITY OF NORTH CAROLINA.

UNC Weight Research Program
www.uncweightresearch.org
1700 Martin Luther King Jr. Blvd., Suite 136
Chapel Hill, North Carolina 27599-7294

NONPROFIT ORG
US Postage
PAID

Generic Brochure

Sign up for SNAP

SNAP is a no-fee research study that provides 18 to 35-year olds with tools to prevent weight gain. Modest weight loss can be expected.

- no fads
- no gimmicks
- no fees

NONPROFIT ORG
US Postage
PAID

UNC Weight Research Program
www.uncweightresearch.org
1700 Martin Luther King Jr. Blvd., Suite 136
Chapel Hill, North Carolina 27599-7294

www.snapaverage.org

THE UNIVERSITY of NORTH CAROLINA at CHAPEL HILL

National Institutes of Health

The **odds are you'll gain 30 pounds** between the ages of 18 and 35.

Beat the odds.

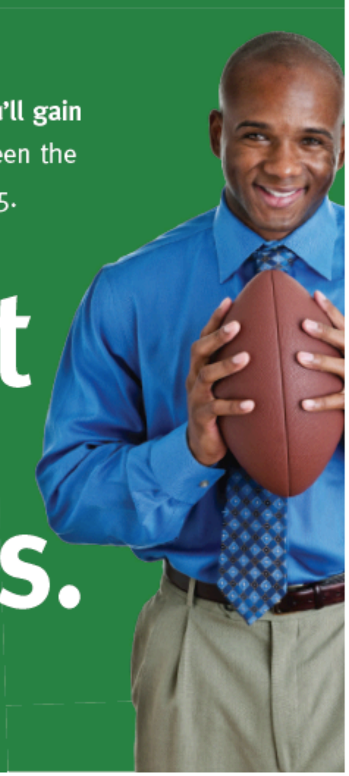

SNAP Healthy Weight Program

STUDY OF NOVEL APPROACHES TO PREVENTION

Want a winning strategy for a healthier future? Join SNAP, a no-fee healthy weight control program.

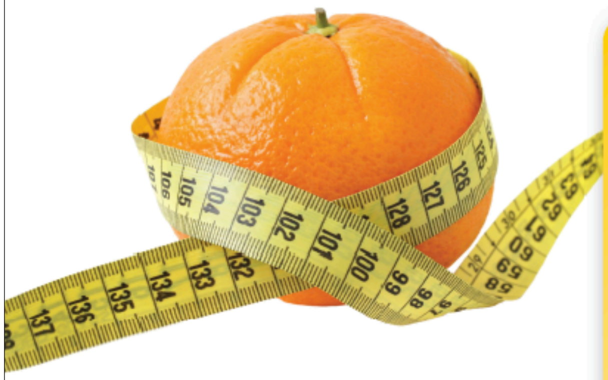

Funded by the the National Institutes of Health and brought to you by leading researchers at the University of North Carolina at Chapel Hill, Miriam Hospital/ The Warren Alpert Medical School of Brown University, and Wake Forest University, SNAP is a cutting-edge research study that teaches strategies for healthier lifestyles and prevention of young adult weight gain which can have a negative impact on health and well-being.

What are the benefits of participating?

In addition to small monetary incentives for completing study related visits, the following will be provided to you free of charge for your participation in SNAP. In a health care setting, these could cost you thousands of dollars and are often not covered by insurance:

- Expertise of weight control professionals (nutritionists, exercise physiologists, physicians, health educators, psychologists, nurses) who will help you to adopt strategies for weight control.
- Personalized feedback on your blood pressure, percent body fat, cholesterol, glucose, insulin and energy expenditure periodically throughout the study.
- Detailed analysis of your dietary intake and physical activity periodically throughout the program.

You can also learn and adopt strategies that will help you to prevent dangerous weight gain and promote a healthier lifestyle for today and the future which may mean:

- Preventing obesity
- Preventing chronic diseases
- Being able to fit in the same size in the coming years
- Healthier habits for better health and fitness
- **A body and lifestyle that makes you proud!**

"This program helped me think critically about what I was eating and the choices I make. Rather than thinking of a "diet," I just consider my options every day. It's OK to eat pizza, ice cream, chips, etc. but that means that I just have to be healthier at the next several meals. It sounds clichéd, but this program taught me the importance of lifestyle changes."

–Sarah N.

Am I eligible for SNAP?

You may be eligible if you are:

- Between the ages of 18 and 35
- Normal to moderately overweight (BMI 21-30, use the BMI calculator at **snapaverage.org** to find your BMI)
- Living within easy driving distance of Raleigh-Durham-Chapel Hill, NC areas or the Providence, RI
- Willing to participate in this important research study

Additional eligibility criteria apply.

For more information, visit us at www.snapaverage.org or call (919) 966-5852

Targeted Brochure

Sign up for SNAP

SNAP is a no-fee research study that provides 18 to 35-year olds with tools to prevent weight gain. Modest weight loss can be expected.

- no fads
- no gimmicks
- no fees

NONPROFIT ORG
US Postage
PAID

UNC Weight Research Program
www.uncweightresearch.org
1700 Martin Luther King Jr. Blvd., Suite 136
Chapel Hill, North Carolina 27599-7294

SNAP Healthy Weight Program
STUDY OF NOVEL APPROACHES TO PREVENTION

www.Snap4men.org

THE UNIVERSITY of NORTH CAROLINA at CHAPEL HILL

National Institutes of Health

The **odds are you'll gain 30 pounds** between the ages of 18 and 35.

MEN: Beat the odds.

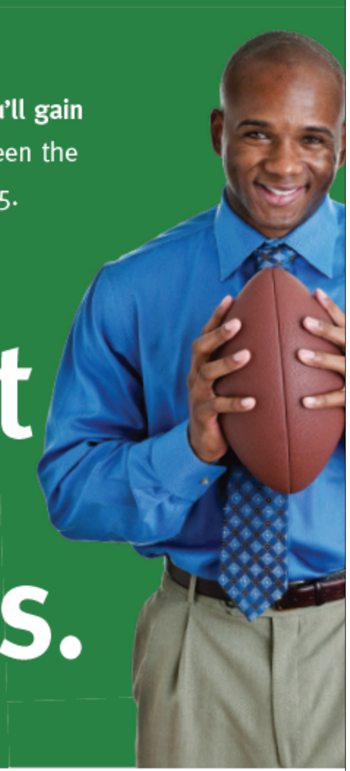

SNAP Healthy Weight Program
STUDY OF NOVEL APPROACHES TO PREVENTION

Want a winning strategy for a healthier future? Join SNAP, a no-fee healthy weight control program.

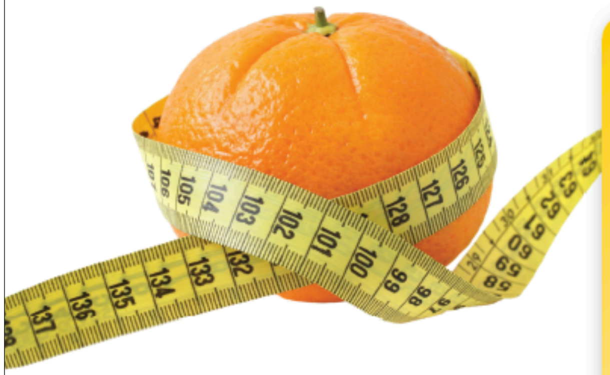

Funded by the the National Institutes of Health and brought to you by leading researchers at the University of North Carolina at Chapel Hill, Miriam Hospital/ The Warren Alpert Medical School of Brown University, and Wake Forest University, SNAP is a cutting-edge research study that teaches strategies for healthier lifestyles and prevention of young adult weight gain which can have a negative impact on health and well-being.

What are the benefits of participating?

In addition to small monetary incentives for completing study related visits, the following will be provided to you free of charge for your participation in SNAP. In a health care setting, these could cost you thousands of dollars and are often not covered by insurance:

- Expertise of weight control professionals (nutritionists, exercise physiologists, physicians, health educators, psychologists, nurses) who will help you to adopt strategies for weight control.
- Personalized feedback on your blood pressure, percent body fat, cholesterol, glucose, insulin and energy expenditure periodically throughout the study.
- Detailed analysis of your dietary intake and physical activity periodically throughout the program.

You can also learn and adopt strategies that will help you to prevent dangerous weight gain and promote a healthier lifestyle for today and the future which may mean:

- Preventing obesity
- Preventing chronic diseases
- Being able to fit in the same size in the coming years
- Healthier habits for better health and fitness
- **A body and lifestyle that makes you proud!**

"This program helped me think critically about what I was eating and the choices I make. Rather than thinking of a "diet," I just consider my options every day. It's OK to eat pizza, ice cream, chips, etc. but that means that I just have to be healthier at the next several meals. It sounds clichéd, but this program taught me the importance of lifestyle changes."

–Sarah N.

Am I eligible for SNAP?

You may be eligible if you are:

- Between the ages of 18 and 35
- Normal to moderately overweight (BMI 21-30, use the BMI calculator at **snap4men.org** to find your BMI)
- Living within easy driving distance of Raleigh-Durham-Chapel Hill, NC areas or the Providence, RI
- Willing to participate in this important research study

Additional eligibility criteria apply.

For more information, visit us at www.snap4men.org or call (919) 966-5852
